# Non-linear associations between cardiovascular metabolic indices and metabolic-associated fatty liver disease: A cross-sectional study in the US population (2017–2020)

**DOI:** 10.1515/biol-2022-0947

**Published:** 2024-09-10

**Authors:** Meimei Xu, Sibo Han, Qiaomei Wu, Shihong Ma, Huiying Cai, Mengqi Xue, Fengling Liu, Xiaozhen Xiao, Xiaoshuang Chen, MeiZhen Lin

**Affiliations:** The Second Affiliated Hospital of Guangzhou University of Chinese Medicine, Guangdong Provincial Hospital of Chinese Medicine, Guangzhou, 510120, Guangdong, China; Hunan University of Chinese Medicine, Changsha, 410208, Hunan, China; The Second Clinical College of Guangzhou University of Chinese Medicine, Guangzhou, Guangdong, 510120, Guangdong, China; Guangzhou Jiangnan Foreign Language School, Guangzhou, 510120, Guangdong, China

**Keywords:** NHANES, cardiometabolic index, metabolic-associated fatty liver disease, statistics, relationship, early diagnosis

## Abstract

The cardiometabolic index (CMI) is an emerging and effective indicator for predicting the presence of metabolic-associated fatty liver disease (MAFLD). This study aims to investigate the relationship between CMI and MAFLD using data from NHANES 2017–2020. In this cross-sectional study, a total of 3,749 subjects were included. The study conducted a thorough analysis of CMI with three multivariate logistic regression models, subgroup analyses, and restricted cubic splines (RCS) were utilized. Using multifactorial logistic regression as the primary method of analysis, we found that a higher CMI was also significantly associated with an increased risk of MAFLD (OR = 1.45, 95% CI (1.05–2.01)). This result was further visualized by the RCS curve: There was a non-linear positive correlation between CMI and MAFLD incidence (the turning point is CMI = 0.4554). These findings were strongly reinforced by subsequent subgroup and sensitivity analyses. There is a robust positive relationship between the CMI and the risk of MAFLD, providing valuable clinical benefits for early detection and screening of MAFLD. It is important to highlight the presence of a non-linear association between CMI and MAFLD, with an inflection point identified at CMI = 0.4554.

## 1 Introduction

Metabolic-associated fatty liver disease (MAFLD) is a prevalent global health issue affecting over one-third of the world’s population, with rates ranging from 32.94 to 44.95% [1,2]. It is characterized by excessive fat accumulation in the liver and metabolic dysfunction, demonstrated by symptoms of overweight or obesity and insulin resistance [3]. In 2020, an international expert panel confidently recommended replacing the term “non-alcoholic fatty liver disease” with “MAFLD.” This decisive change offers a broader perspective by encompassing various causes of fatty liver diseases and makes it easier to identify individuals at risk of developing liver conditions [4,5,6]. MAFLD is strongly associated with the development of hepatocellular carcinoma and cirrhosis, and it significantly increases the likelihood of cardiovascular diseases [7]. Therefore, prompt diagnosis and early intervention are crucial to effectively alleviate the burden of MAFLD.

Liver biopsy is considered the “gold standard” for diagnosing MAFLD [8]. However, its use in clinical practice is limited due to its invasiveness, complexity, cost, and challenges with patient compliance. Although ultrasound is a common diagnostic tool, mild steatosis is rare and can be influenced by equipment quality and operator skill [8]. Conversely, computed tomography has good diagnostic sensitivity, but it is limited by radiation exposure. Magnetic resonance imaging (MRI) is the preferred method for assessing liver fat content in clinical and research settings due to its non-invasive, precise, and reproducible nature. Although MRI may have some specificity issues and can be expensive, it remains the most reliable option available [8,9]. To detect MAFLD early, it is essential to investigate a low-cost, practical, and straightforward secondary diagnostic tool. Hepatic steatosis, a key feature of MAFLD [10], is characterized by an excess buildup of triglycerides and cholesterol in hepatocyte lipid droplets caused by the accumulation of visceral fat. However, central obesity and insulin resistance are primarily associated with visceral fat buildup and hepatic steatosis [11]. Body fat indicators such as body mass index (BMI), waist circumference, waist-to-height ratio, transaminases, fatty acids, serum triglyceride, total cholesterol (TG), high-density lipoprotein cholesterol (HDL-C), and the TG/HDL-C ratio are crucial in evaluating MAFLD due to their strong correlation with the condition [12,13]. The TG/HDL-C ratio predicts non-alcoholic fatty liver disease and is associated with insulin resistance, abdominal obesity, metabolic disturbances, and cardiometabolic risks [14].

The cardiometabolic index (CMI) is a highly effective method for assessing the distribution and dysfunction of visceral fat [15]. Numerous studies have demonstrated the high predictive power of CMI for MAFLD in both Chinese [16] and US [17] populations. CMI was found to be an effective tool for assessing the risk of NAFLD in patients with T2D, who are at elevated risk for liver-related complications [18]. Furthermore, CMI was identified as an effective monitoring method, a finding that was corroborated by a longitudinal cohort study from China [19].

Therefore, CMI is a reliable and valuable tool for assessing MAFLD risk. Numerous studies have demonstrated the high predictive power of CMI for MAFLD in both Chinese [16,18,19] and US populations [17]. Although there is a lack of relevant research, the findings suggest that CMI has potential clinical use, and further evidence is needed to support this claim. This study aims to comprehensively explore the link between CMI and MAFLD using a large sample of adults in the United States.

## 2 Methods and materials

### 2.1 Study population

**Figure 1:**
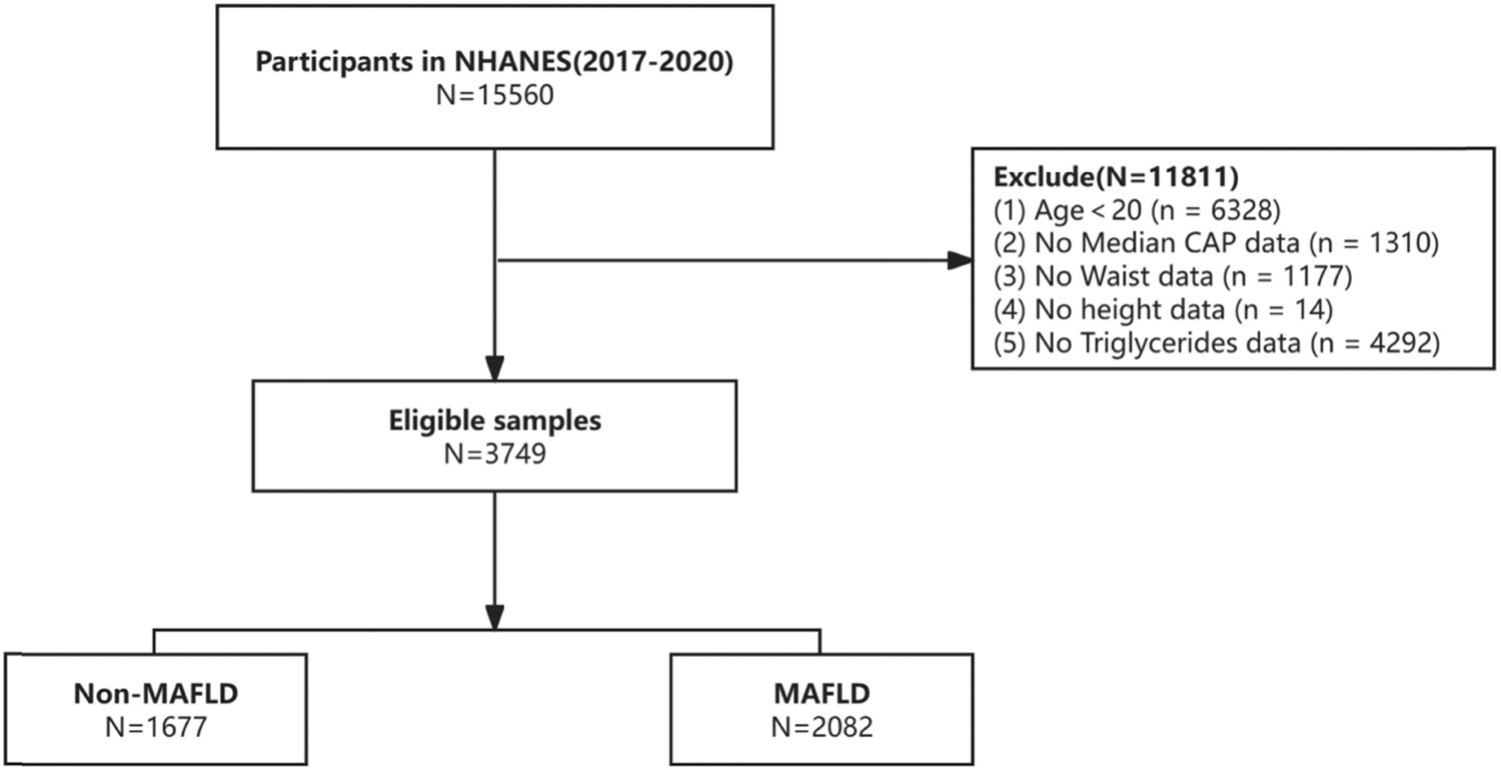
Flowchart of the sample selection from NHANES 2017–2020.

The NHANES database is an annual nationwide survey that has been conducted on the nutrition and health of the US population since 1999. It utilizes sophisticated sampling methods, including multistage and stratified sampling, to specifically target minors and older adults. For more detailed information, you can visit the official website at https://www.cdc.gov/nchs/nhanes/index.htm. This website offers a comprehensive overview of the NHANES survey design, which received approval from the National Center for Health Statistics Ethical Review Board before data collection, with all participants providing informed consent. The survey included household questionnaires, telephone interviews, and physical examinations carried out by medical professionals and trained staff to gather data. Data for this cross-sectional study were extracted from the NHANES database covering the years 2017 to 2020. Exclusions were made for 6,328 participants under the age of 20,1310 participants with missing median cap data, 1,177 participants with missing waist data, 14 participants with missing height data, and 4,292 participants with missing triglyceride data. In total, a total of 3,749 individuals were included in this study (Figure 1).

### 2.2 CMI

The CMI was calculated using blood samples collected along with anthropometric measurements. Stringent laboratory tests and assessments of key indicators were carried out in accordance with standardized sampling procedures to ensure the accuracy and consistency of the data. Blood samples were usually gathered either on survey vehicles or at assigned collection points, then processed and analyzed in the laboratory. Certified healthcare professionals measurede participants’ height and waist circumference at mobile screening facilities [20].

### 2.3 MAFLD

MAFLD is diagnosed if transient elastography shows hepatic steatosis together with one of the following: metabolic abnormalities, type 2 diabetes, or overweight/obesity [21]. Significant hepatic steatosis is indicated by a controlled attenuation parameter (CAP) exceeding 248 dB/m [22,23]; CAP is widely used worldwide as a tool to assess the degree of fatty liver degeneration [23]. A BMI above 25 kg/m^2^ indicates overweight or obesity, while diabetes is diagnosed with an FBG level of 7.0 mmol/L, a glycosylated hemoglobin level of 6.5%, a confirmed diagnosis by healthcare providers, or the use of diabetes medication. Metabolic abnormalities are diagnosed in lean or normal-weight individuals when two or more of the following factors are present: (1) waist circumference over 102 cm for males or 88 cm for females; (2) blood pressure of 130/85 mmHg or taking antihypertensive medication; and (3) serum triglyceride levels exceeding 150 mg/dL (or 1–70 mmol/L) or receiving specific drug therapy. (4) Low levels of HDL-C are defined as <40 mg/dL for males and <50 mg/dL for females or if the patient is receiving specific medication. (5) Pre-diabetes is indicated by FPG levels between 100 and 125 mg/dL. An individual is considered to have pre-diabetes if their fasting plasma glucose (FPG) levels are between 100 and 125 mg/dL, or if post-load blood glucose levels are between 140 and 199 mg/dL 2 h after ingestion, or if HbA1c levels are between 5 and 7%, or if the homeostasis model assessment of insulin resistance score is above 2, or if high-sensitivity C-reactive protein (HsCRP) is elevated (>2 mg/l).

### 2.4 Covariates

The covariates considered in this study were gender, age, race, education level, marital, poverty-to-income ratio (PIR), alcohol consumption, smoking, physical activity (PA), diabetes, hypertension, and depression. Additionally, median capillary blood glucose levels, and serum uric acid (SUA) levels were measured along with fasting blood glucose levels and glycosylated hemoglobin. Homeostasis model assessment of insulin resistance (HOMA-IR) and serum urea nitrogen were also assessed.

Race categories included non-Hispanic white, non-Hispanic black, Mexican American, other Hispanic, and other racial groups. Education level was categorized as less than high school or high school and above. Marital status was divided into married and unmarried individuals. PIR was defined as low level (PIR < 1.30), medium level (1.30 ≤ PIR < 3.50), and high level (≥3.50) [24]. Nondrinkers were defined as individuals who had not consumed alcohol in the past 12 months or throughout their lifetime. Smokers were defined as those who had smoked more than 100 cigarettes in their lifetime. PA was classified as low (<600 min/week), moderate (600–8,000 min/week), and high (≥8,000 min/week) based on metabolic equivalent task minutes per week [25]. Hypertension was defined as systolic blood pressure (SBP) ≥130 mmHg, diastolic blood pressure (DBP) ≥80 mmHg, self-reported hypertension or use of antihypertensive medications, or a physician’s diagnosis of hypertension. Depression severity was assessed using the Patient Health Questionnaire-9, a reliable measure that evaluates the frequency of depressive symptoms over the past 2 weeks. The questionnaire consists of nine questions scored from “0” to “3,” with a total score ranging from 0 to 27. A score of 10 or higher indicates the presence of depression [26].

The laboratory tests also encompassed alanine aminotransferase (ALT), aspartate aminotransferase (AST), blood urea nitrogen (BUN), uric acid, serum potassium, serum iron, serum globulin, and red blood cell distribution width. The physical examination comprised SBP, DBP, BMI, waist circumference, and waist-to-height ratio.

### 2.5 Statistical methods

Continuous variables are presented as mean with standard deviation (SD). Categorical variables are shown as frequencies or percentages. Baseline characteristics were analyzed using the *t*-test or one-way analysis of variance for continuous variables, and the chi-square test or Fisher’s exact test for categorical variables. Missing values were managed by simple replacement. In categorical variables, missing values were identified as unknown. The mean value was used for filling in missing values following a normal distribution. In cases of skewed distribution of missing values, the mode was employed for replacement. Moreover, sensitivity analyses were carried out by conducting analyses with all missing values excluded (Table S1) and multiple imputations of missing values (Table S2) to ensure the robustness of our findings, which are detailed in the Supplementary Material. Logistic regression analysis was used to investigate the association between CMI and MAFLD. The results were reported as the odds ratio and corresponding 95% confidence interval. Important variables from univariate and multivariate logistic regression were included for covariate screening.

Logistic regression analysis was used to investigate the association between the CMI and MAFLD. The study reported the results in terms of odds ratios and 95% confidence intervals, which shows the level of confidence in the results. Covariates were selected based on their significance in univariate logistic regression analysis as well as clinical expertise. The covariates considered included age, gender, race, education status, marital status, PIR, alcohol status, smoking status, PA, diabetes status, hypertension status, depression status, median cap, SUA levels, FPG, HbA1c, homeostasis model assessment of insulin resistance, and serum urea nitrogen (BUN) levels.

In the multivariate logistic regression analysis, categorical variables were transformed into quartiles to further explore the association between CMI and the occurrence of MAFLD. The first model served as the initial unadjusted model, while the second model was adjusted for various factors, including age, race, gender, education status, PIR, marital status, alcohol consumption status, smoking status, PA, diabetes status, hypertension status, and depression status.

Moreover, the third model included further adjustments for median cap, SUA, FPG, HbA1c, HOMA-IR, and BUN building upon the second model. Restricted cubic splines were used to confidently explore the potential non-linear correlation between CMI and the occurrence of MAFLD. The evaluation of linearity and investigation of any curvilinear relationship between CMI and MAFLD were conducted while considering the variables in the third model. The association threshold between CMI and MAFLD was determined through an analysis that utilized smoothing techniques in a two-segment logistic regression model. The third model was adjusted for these variables. The inflection point was identified through likelihood ratio tests and bootstrap regression analysis.

Subgroup analyses were performed to evaluate the strength of the results by considering various factors such as gender, age, race, alcohol consumption, smoking, diabetes, hypertension, depression, and BMI. Additionally, the potential impact of variables such as gender, age, marital status, education level, drinking status, smoking status, diabetes status, hypertension status, depression status, and BMI on the relationship between CMI and MAFLD was assessed. Multivariate logistic regression analysis was used to assess heterogeneity between subgroups, and likelihood ratio tests were used to assess interactions between subgroups and CMI.

The statistical software R 3.3.2 was utilized for the analyses, in addition to Free Statistics software version 1.9 and www.R-project.org. The descriptive study encompassed all participants, and statistical significance was assessed using a two-tailed test with a *p*-value threshold of <0.05.

## 3 Results

### 3.1 Characteristics of participants

In total, 3,749 participants participated in the trial, with an average age of 52 years and 48.5% male. Among the participants, 12.8% were Mexican American, 10.2% were of Other Hispanic descent, and 33.9% were Non-Hispanic White. Non-Hispanic blacks made up 25.3% of the group. The overall prevalence of MAFLD was determined to be 55.5%. Table 1 displays the baseline characteristics of the groups stratified by MAFLD. Patients with MAFLD, when compared to those without, were generally older and more likely to be male. They also had a higher frequency of being married and smoking, as well as a greater prevalence of hypertension and depression. Additionally, their SBP, DBP, SUA, BMI, waist circumference, WtHR, HbA1c, hs-CRP, HOMA-IR, ALT, AST, and BUN levels were all significantly higher compared to individuals without MAFLD (all *p* < 0.05).

**Table 1:** Basic characteristics of study participants

Variables	Total (*n* = 3,749)	Non-MAFLD(*n* = 1,667)	MAFLD (*n* = 2,082)	*p*
**Gender**				<0.001
Male	1,820 (48.5)	743 (44.6)	1,077 (51.7)	
Female	1,929 (51.5)	924 (55.4)	1,005 (48.3)	
**Race**				<0.001
Mexican American	481 (12.8)	161 (9.7)	320 (15.4)	
Other Hispanic	381 (10.2)	158 (9.5)	223 (10.7)	
Non-Hispanic White	1,272 (33.9)	525 (31.5)	747 (35.9)	
Non-Hispanic Black	947 (25.3)	501 (30.1)	446 (21.4)	
Other	668 (17.8)	322 (19.3)	346 (16.6)	
**PIR**				0.326
Low	873 (23.3)	402 (24.1)	471 (22.6)	
Middle	1,305 (34.8)	554 (33.2)	751 (36.1)	
High	1,071 (28.6)	483 (29)	588 (28.2)	
**Education**				0.12
<High school	709 (18.9)	294 (17.6)	415 (19.9)	
≥High school	3,038 (81.0)	1,372 (82.3)	1,666 (80)	
**Married**				<0.001
No	1,526 (40.7)	751 (45.1)	775 (37.2)	
Yes	2,219 (59.2)	914 (54.8)	1,305 (62.7)	
**Alcohol**				0.105
No	3,395 (90.6)	1,524 (91.4)	1,871 (89.9)	
Yes	354 (9.4)	143 (8.6)	211 (10.1)	
**PA**				0.366
Low	463 (12.3)	198 (11.9)	265 (12.7)	
Middle	1,682 (44.9)	749 (44.9)	933 (44.8)	
High	676 (18.0)	319 (19.1)	357 (17.1)	
**Smoke**				0.02
No	2,133 (56.9)	986 (59.1)	1,147 (55.1)	
Yes	1,614 (43.1)	680 (40.8)	934 (44.9)	
**Hypertension**				<0.001
No	1,647 (43.9)	925 (55.5)	722 (34.7)	
Yes	2,102 (56.1)	742 (44.5)	1,360 (65.3)	
**Diabetes**				0.083
No	1,293 (34.5)	600 (36)	693 (33.3)	
Yes	2,456 (65.5)	1,067 (64)	1,389 (66.7)	
**Depression**				0.013
No	2,828 (75.4)	1,290 (77.4)	1,538 (73.9)	
Yes	921 (24.6)	377 (22.6)	544 (26.1)	
Median CAP (dB/m)	265.8 ± 60.9	213.4 ± 35.6	307.7 ± 41.4	<0.001
SBP (mmHg)	123.8 ± 18.2	121.3 ± 18.6	125.7 ± 17.7	<0.001
DBP (mmHg)	74.3 ± 11.1	72.3 ± 10.9	75.9 ± 10.9	<0.001
K+ (mmol/L)	4.1 ± 0.4	4.1 ± 0.4	4.1 ± 0.4	0.002
SUA (mg/dL)	5.4 ± 1.5	5.1 ± 1.4	5.7 ± 1.4	<0.001
CMI	0.5 (0.3, 0.8)	0.3 (0.2, 0.5)	0.6 (0.4, 1.0)	<0.001
Age	52.0 (36.0, 64.0)	46.0 (31.0, 63.0)	55.0 (41.0, 65.0)	<0.001
BMI (kg/m^2^)	28.6 (24.8, 33.6)	25.5 (22.5, 29.2)	31.1 (27.5, 36.0)	<0.001
Waist (cm)	99.3 (88.6, 111.5)	90.4 (81.2, 100.1)	106.1 (97.0, 117.5)	<0.001
WHtR	0.6 (0.5, 0.7)	0.5 (0.5, 0.6)	0.6 (0.6, 0.7)	<0.001
FPG (mmol/L)	5.7 (5.3, 6.4)	5.5 (5.2, 5.9)	6.0 (5.6, 6.9)	<0.001
HbA1c	5.6 (5.3, 6.0)	5.4 (5.2, 5.7)	5.7 (5.4, 6.2)	<0.001
HsCRP (mg/L)	2.0 (0.8, 4.3)	1.3 (0.6, 3.2)	2.6 (1.2, 5.2)	<0.001
HOMA-IR	2.6 (1.5, 4.7)	1.7 (1.1, 2.7)	3.7 (2.3, 6.2)	<0.001
RDW	13.6 (13.1, 14.3)	13.5 (13.0, 14.2)	13.6 (13.1, 14.4)	<0.001
ALT (µ/l)	17.0 (13.0, 25.0)	15.0 (12.0, 21.0)	20.0 (14.0, 29.0)	<0.001
AST (µ/l)	19.0 (16.0, 23.0)	18.0 (15.0, 22.0)	19.0 (16.0, 24.0)	<0.001
BUN (mg/dL)	14.0 (11.0, 17.0)	13.0 (11.0, 17.0)	14.0 (11.0, 17.0)	<0.001
Globulins (g/dL)	3.1 (2.8, 3.4)	3.1 (2.8, 3.4)	3.1 (2.8, 3.4)	0.002
Fe (µmol/L)	6.8 (5.1, 10.3)	6.8 (5.1, 10.3)	6.8 (5.1, 10.3)	0.076

PIR, ratio of family income to poverty; PA, physical activity; SBP, systolic blood pressure; DBP, diastolic blood pressure; K+, Serum potassium concentration; SUA, serum uric acid; CMI, cardiometabolic index; BMI, body mass index; WHtR, waist-height ratio; FPG, fasting blood glucose; HbA1c, glycosylated hemoglobin; HsCRP, high-sensitivity C-reactive protein; HOMA-IR, Homeostatic Model Assessment for Insulin Resistance; RDW, red cell distribution width; ALT, alanine aminotransferase; AST, aspartate aminotransferase; BUN, blood urea nitrogen; Fe, serum iron.

### 3.2 The association between CMI and MAFLD

In the univariate analysis, significant associations were found between MAFLD and various factors such as age, gender, race, marital status, alcohol status, smoking status, hypertension status, BMI, waist circumference, waist-height ratio, FPG, glycosylated hemoglobin, hs-CRP, HOMA-IR, AST, ALT, BUN, and SUA, as indicated in Table 2.

**Table 2:** Association between MAFLD and covariates

Variable	OR_95% CI	*p*_value
**CMI**	8.04 (6.53–9.89)	<0.001
**Gender**		
Male (1890)		
Female (1895)	0.75 (0.66–0.85)	<0.001
**Race**		
Mexican American (481)		
Other Hispanic (381)	0.71 (0.54–0.94)	0.016
Non-Hispanic White (1272)	0.72 (0.57–0.89)	0.003
Non-Hispanic Black (947)	0.45 (0.36–0.56)	<0.001
Other (668)	0.54 (0.42–0.69)	<0.001
**PIR**		
Low (873)		
Middle (1305)	1.16 (0.97–1.37)	0.098
High (1701)	1.04 (0.87–1.24)	0.676
**Education**		
<High school (709)		
≥High school (3038)	0.86 (0.73–1.02)	0.075
**Married**		
No (1526)		
Yes (2219)	1.38 (1.21–1.58)	<0.001
**Alcohol**		
No (3395)		
Yes (354)	1.2 (0.96–1.5)	0.106
**PA**		
Low (463)		
Middle (1682)	0.93 (0.76–1.15)	0.498
High (676)	0.84 (0.66–1.06)	0.141
**Smoke**		
No (2133)		
Yes (1614)	1.18 (1.04–1.35)	0.013
**Hypertension**		
No (1647)		
Yes (2102)	2.35 (2.06–2.68)	<0.001
**Diabetes**		
No (1293)		
Yes (2456)	1.13 (0.98–1.29)	0.083
**Depression**		
No (2828)		
Yes (921)	1.21 (1.04–1.41)	0.013
Median CAP (dB/m)	1.12 (1.11–1.13)	<0.001
SBP (mmHg)	1.01 (1.01–1.02)	<0.001
DBP (mmHg)	1.03 (1.02–1.04)	<0.001
K+ (mmol/L)	1.33 (1.11–1.6)	0.002
SUA (mg/dL)	1.36 (1.3–1.43)	<0.001
Age	1.02 (1.01–1.02)	<0.001
BMI (kg/m^2^)	1.17 (1.16–1.19)	<0.001
Waist (cm)	1.08 (1.07–1.08)	<0.001
WHtR	71371.34 (29191.98–174495.47)	<0.001
FPG (mmol/L)	1.51 (1.42–1.61)	<0.001
HbA1c	1.82 (1.66–2)	<0.001
HsCRP (mg/L)	1.04 (1.02–1.05)	<0.001
HOMA-IR	1.23 (1.19–1.26)	<0.001
RDW	1.04 (0.99–1.09)	0.106
ALT (µ/l)	1.03 (1.03–1.04)	<0.001
AST (µ/l)	1.01 (1–1.02)	0.001
BUN (mg/dL)	1.02 (1.01–1.03)	0.003
Globulins (g/dL)	1.25 (1.07–1.45)	0.004
Fe (µmol/L)	0.99 (0.97–1)	0.033

PIR, ratio of family income to poverty; PA, physical activity; SBP, systolic blood pressure; DBP, Diastolic blood pressure; K+, serum potassium concentration; SUA, serum uric acid; CMI, cardiometabolic index; BMI, body mass index; WHtR, waist-height ratio; FPG, fasting blood glucose; HbA1c, glycosylated hemoglobin; HsCRP, high-sensitivity C-reactive protein; HOMA-IR, homeostatic model assessment for insulin resistance; RDW, red cell distribution width; ALT, alanine aminotransferase; AST, aspartate aminotransferase; BUN, blood urea nitrogen; Fe, serum iron.

Furthermore, there was a significant association between MAFLD and the CMI when evaluated as a continuous variable. When the CMI was stratified into quartile groups, the correlation with MAFLD remained statistically significant. To better account for possible confounding variables, we performed additional logistic multivariate regression analysis and constructed three models to explore the relationship between CMI and MAFLD (Table 3).

**Table 3:** Association between CMI and MAFLD in multiple models

	Model Ⅰ		Model Ⅱ		Model Ⅲ	
	OR (95% CI)	*P* value	OR (95% CI)	*P* value	OR (95% CI)	*P* value
CMI (3749)	8.04 (6.53–9.89)	<0.001	6.8 (5.49–8.44)	<0.001	1.44 (1.04–1.99)	0.026
CMI*						
＜0.4554 (1855)	1 (Ref)		1 (Ref)		1 (Ref)	
≥0.4554 (1894)	4.73 (4.12–5.43)	<0.001	4.24 (3.66–4.92)	<0.001	1.2 (0.89–1.62)	0.233
CMI**						
Q1 (937)	1 (Ref)		1 (Ref)		1 (Ref)	
Q2 (937)	2.52 (2.07–3.05)	<0.001	2.26 (1.85–2.76)	<0.001	2.03 (1.37–3.01)	<0.001
Q3 (937)	5.32 (4.37–6.49)	<0.001	4.76 (3.87–5.86)	<0.001	1.58 (1.06–2.37)	0.026
Q4 (938)	12.24 (9.82–15.25)	<0.001	10.51 (8.32–13.28)	<0.001	2.07 (1.31–3.27)	0.002

CMI^*^, CMI is treated as a continuous variable; CMI^**^, CMI is treated as a dichotomous variable; CMI^***^, CMI is treated as a quartile variable; Model Ⅰ, non-adjusted model; Model Ⅱ, adjusted for age, gender, race, education, PIR, married, smoke, alcohol, PA, hypertension, diabetes, depression; Model Ⅲ, Model Ⅱ + median CAP, SUA, BUN, FPG, HbA1c, HOMA-IR.

In Model 1, the initial analysis showed a positive correlation between CMI and MAFLD, with an odds ratio of 8.04 (95% CI: 6.53–9.89). When CMI was divided into quartiles, with Q1 as the baseline, a 0.1-unit rise in CMI in Q2 was associated with a 15.2% higher likelihood of MAFLD (95% CI: 2.07–3.05%). The Q3 group saw a 43.2% rise (95% CI: 2.07–3.05) in MAFLD occurrence for every 0.1-unit increase in CMI, while a 0.01-unit increase in CMI in Q4 resulted in an 11.24% increase (95% CI: 9.82–15.25) in MAFLD prevalence.

Adjusting for multiple variables in Model 2, such as gender, age, race, education status, marital status, PIR, alcohol status, smoking status, PA, diabetes, hypertension, and depression, revealed a positive association between the CMI and MAFLD (OR = 6.83, 95% CI: 5.51–8.47). Analysis of the quartiles of the CMI showed that compared to the Q1 group, each 0.1 increment in CMI within the Q2 group was linked to a 12.7% higher likelihood of developing MAFLD (95% CI: 1.86–2.77). Similarly, in the Q3 group, each 0.1 increase in CMI was associated with a 37.6% higher incidence of MAFLD (95% CI: 3.87–5.85). Within the Q4 group, a 0.01 rise in CMI corresponded to a significant 9.57% increase in MAFLD incidence (95% CI: 8.37–13.36).

In Model 3, additional adjustments were made for median cap, SUA, FPG, HbA1c, HOMA-IR, and BUN compared to Model 2. A positive association between CMI and MAFLD was found in the study (OR = 1.45, 95% CI (1.05–2.01)). When stratifying the CMI into quartiles, it was observed that compared to the lowest quartile (Q1), each 0.1 increment in CMI within the second quartile (Q2) was associated with an 11.2% increase in the incidence of MAFLD (95% CI: 1.44–3.14). Similarly, in the third quartile (Q3), a 0.1 increase in CMI resulted in a 6.9% rise in the occurrence of MAFLD (95% CI: 1.13–2.52). Within the highest quartile (Q4), every 0.1 increase in CMI was linked to an 11.5% increase in the incidence of MAFLD (95% CI: 1.36–3.4).

**Figure 2:**
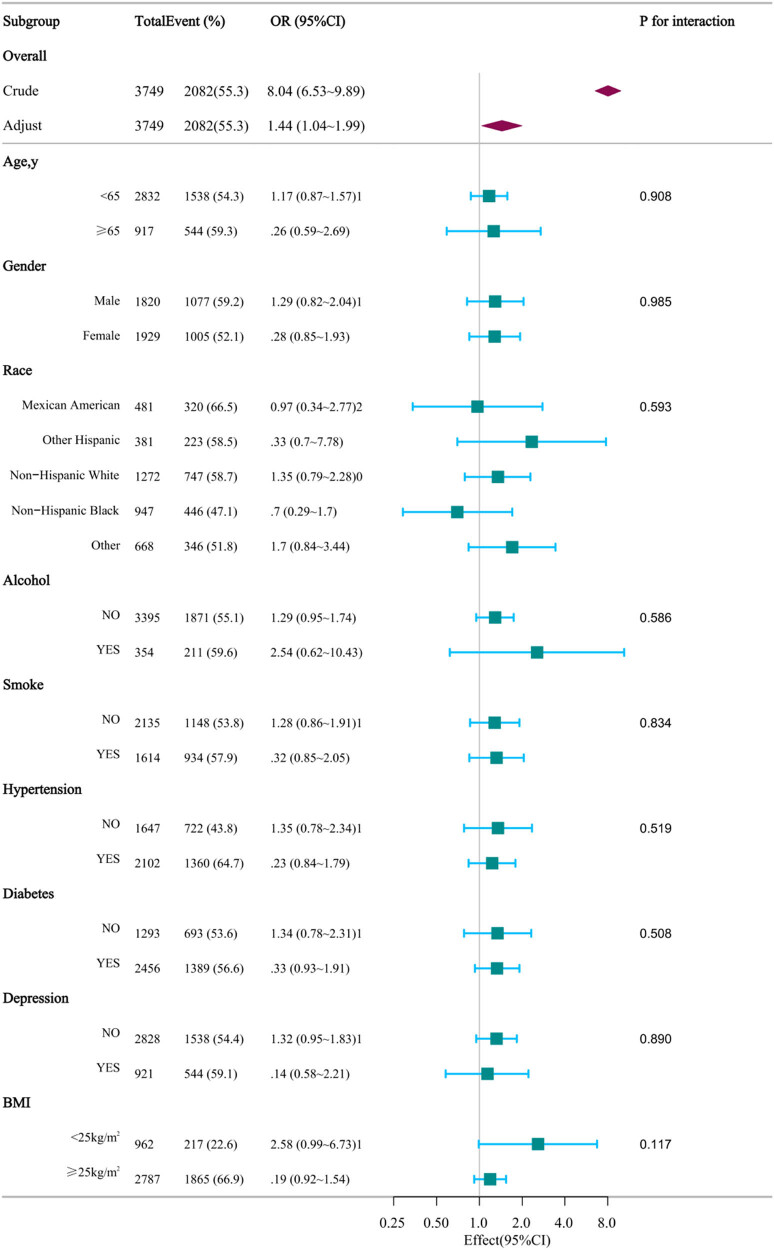
Association Between CMI and MAFLD. Each stratification was adjusted for age, gender, race, education, PIR, married, smoking, alcohol, PA, hypertension, diabetes, depression, median CAP, SUA, BUN, FPG, HbA1c, and HOMA-IR. The stratification factor itself was excluded from the adjustments. OR are represented by squares with horizontal lines indicating 95% CI. Overall ORs are represented by diamonds with outer points indicating 95% CI.

The RCS (restricted cubic spline) of CMI and MAFLD was plotted to assess linearity and investigate the curved connection between CMI and MAFLD after accounting for variables in Model 3. As shown in Figure 2, a non-linear correlation between CMI and MAFLD was detected (P for non-linearity = 0.005), suggesting that their association is not purely linear. At CMI = 0.4554, we identified a turning point in the relationship between CMI and the risk of MAFLD.

Consequently, we divided CMI into two categories and conducted multivariate logistic regression analysis, as shown in Table 3. Using CMI < 0.4554 as the baseline group, in model 1, a 0.1 increase in CMI above 0.4554 showed a substantial 37.3% rise (95% CI: 4.12–5.43) in MAFLD incidence. In model 2, individuals with CMI above 0.4554 experienced a notable 32.4% increase (95% CI: 3.66–4.92) in MAFLD incidence for each 0.1 elevation in CMI level beyond the inflection point. These findings were statistically significant, with a significance level of *p* ≤ 0.05. Nevertheless, in model 3, among the subset with CMI0.4554, there was a 2.3% rise (95% CI: 0.92–1.66) in the occurrence of MAFLD for each 0.1 increase in CMI. However, this result was not statistically significant (*p* > 0.05), potentially due to the significant difference in sample size between the two subsets.

### 3.3 Subgroup analyses

**Figure 3:**
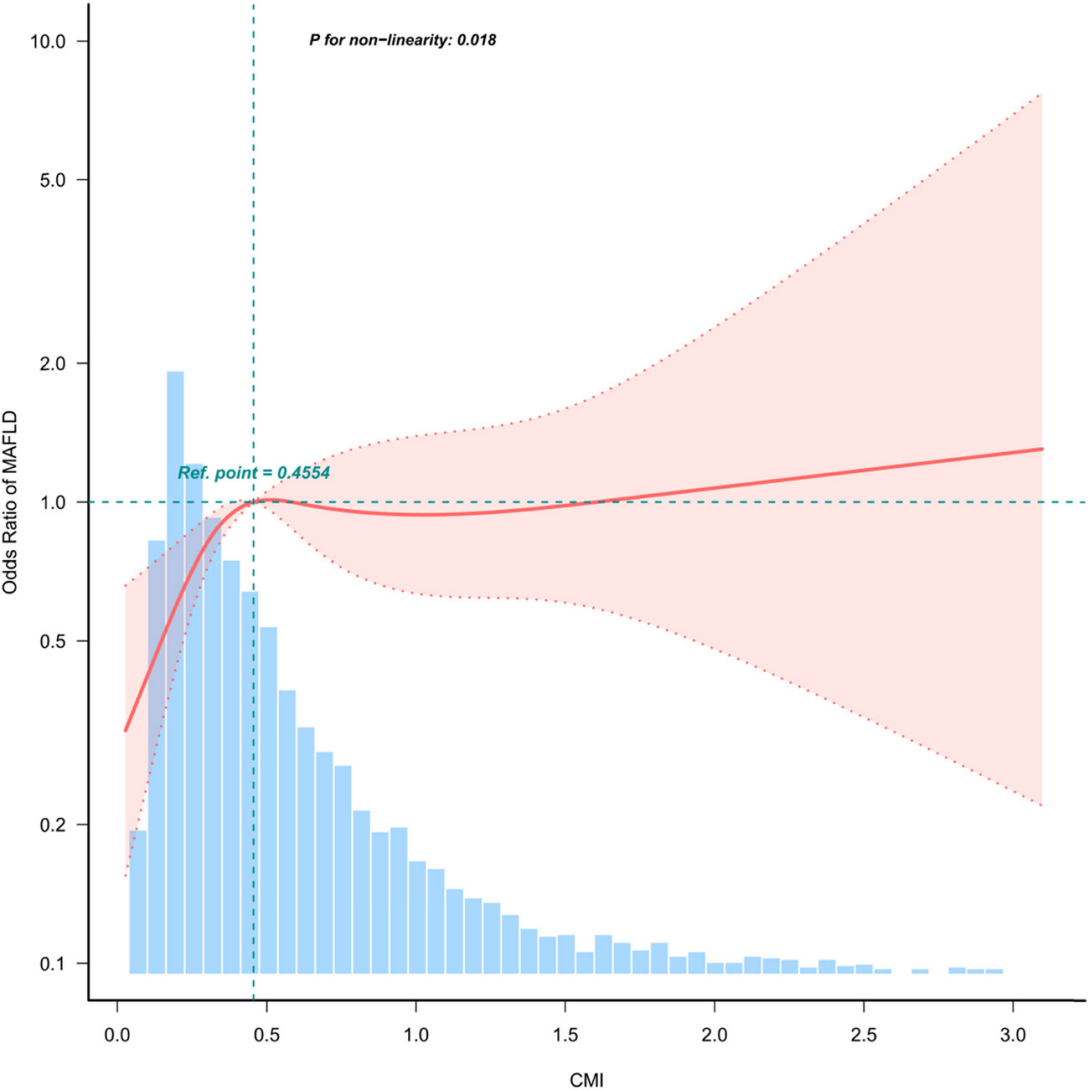
The non-linear association between the CMI and MAFLD. The non-linear relationships between CMI and MAFLD were examined. The solid red line represents the smoothed curve fit depicting the associations between these variables. The blue bands indicate the 95% confidence interval derived from the fitted model (the inflection point was CMI = 0.4554; *p* for non-linearity = 0.018).

In order to validate the findings of logistic multivariate regression model 3, we performed subgroup analyses based on various factors, including age, gender, BMI, race, PIR, education status, marital status, alcohol, PA, smoking status, hypertension, diabetes, and depression. This was carried out to explore potential interactions between CMI and MAFLD, as well as to identify any biases that may be present in different population groups. Importantly, no significant interactions were detected within any subgroup (Figure 3), confirming the consistent and dependable results across all subgroup analyses (*p* > 0.05).

### 3.4 Sensitivity analyses

After exclusion of participants with missing covariates, a total of 3,237 participants were included for analysis in the multivariable logistic regression model 3 (please refer to Table S1). The results showed a positive relationship between CMI and MAFLD (OR = 1.89, 95% CI: 0.93–3.86). When examining the CMI based on quartiles, using Q1 as the reference category, each 0.1 increase in CMI within Q2 was linked to a 9.5% higher likelihood of MAFLD (95% CI: 1.47–2.58). In the Q3 category, every 0.1 increase in CMI correlated with a 5.6% increased probability of MAFLD (95% CI: 1.14–2.13). Similarly, within the Q4 group, each 0.1 rise in CMI was associated with a significant 6.3% increase in the likelihood of MAFLD (95% CI: 1.11–2.39). After conducting multiple imputations to address missing covariates, a total of 18,745 participants were included in multivariate logistic regression model 3 (Table S2). The findings showed a positive association between CMI and MAFLD, with an OR of 1.18 and a 95% CI of 0.87–1.6. When examining CMI in quartiles with Q1 as the baseline, each 0.1 increment in CMI in Q2 was linked to a 5.9% increase in MAFAD incidence (95% CI: 0.84–3). Notably, in the Q3 group, a 0.1 rise in CMI corresponded to a 4.8% upsurge in MAFAD incidence (95% CI: 0.74–2.94), while a 0.01 unit increase in CMI within the Q4 category was associated with a substantial 14.6% elevation in MAFAD risk, with a confidence interval spanning from 1.05 to 5.76.

## 4 Discussion

In our cross-sectional analysis using the NHANES 2017–2020 dataset, we have conclusively shown a direct link between the CMI and MAFLD. When stratifying the CMI into quartiles, this connection becomes even more apparent. It is worth noting that a complex curve is present, with a turning point at CMI = 0.4554, suggesting that the relationship between the CMI and MAFLD is not purely linear. The reliability of these results has been confirmed through additional subgroup and sensitivity analyses, underscoring their considerable clinical importance.

The results of a cross-sectional study conducted in China illustrate a strong positive correlation between CMI and the likelihood of MAFLD, underscoring its efficacy as a dependable predictor for early detection and screening of MAFLD [27]. This emphasizes the substantial clinical utility that CMI offers in this context. In Duan’s research [16], a notable interaction between CMI and the risk of MAFLD was noted in relation to gender, age, and BMI. However, this observation was not reaffirmed in our study, possibly due to differences in the demographics and sample sizes of the study groups.

A retrospective study carried out in Dongxing Gu demonstrated that CMI can serve as a convenient screening tool for MAFLD, as each standard deviation increase in CMI was linked to a heightened risk of MAFLD [28] (OR = 3.26, 95% CI: 2.36–4.51 for females OR = 2.72, 95% CI: 2.35–3.15 for males). Moreover, the prevalence of MAFLD showed an increasing trend corresponding to higher CMI quartiles, further supporting the role of CMI in assessing MAFLD risk. The results of a cohort study conducted in a Thai HIV population indicate an association between MAFLD and augmentation in epicardial fat mass, suggesting a notable relationship between MAFLD and other metabolic disorders [29].

While BMI and waist circumference (WC) are commonly used to assess obesity, they have limitations in effectively distinguishing between complex body metabolic conditions. Using data from NHANES 2017–2018, a study by Li explored the relationship between CMI and MAFLD and found that CMI showed good diagnostic performance for MAFLD [17], especially with a notable gender-specific interaction. Although the exact mechanisms linking CMI and MAFLD are not fully understood, our results are supported by strong biological evidence. Previous research has consistently shown that MAFLD significantly increases the risk of cardiovascular diseases [30,31]. Studies have also indicated a strong link between TG/HDL-C and insulin resistance, obesity, and metabolic disorders, emphasizing its robust predictive value for NAFLD [32]. WHtR, as a marker of abdominal obesity, is closely related to lipid content and distribution [14], outperforming WC and BMI in accurately identifying NAFLD [14,33]. Our study further supports the idea that CMI is strongly associated with the risk of developing MAFLD. Building on previous research, CMI has great potential in evaluating visceral fat distribution and related health issues.

MAFLD involves a complex interplay of factors, such as insulin resistance linked to metabolic syndrome, lipotoxicity from excessive lipid accumulation, inflammation-induced hepatic damage due to infiltrating cells, and subsequent activation of hepatic stellate cells leading to fibrosis. Insulin resistance is crucial in the onset and advancement of MAFLD [34].

Previous research has noted notable variations in gender and obesity categories during subgroup analysis. Nevertheless, our study did not find the same disparities. The outcomes of our subgroup investigations, which involved eliminating individuals with incomplete covariate data and employing multiple imputations (refer to the Supplementary Material), aligned with the general findings of our research. It is important to highlight that a significant number of individuals with MAFLD in clinical settings (ranging from 6 to 20%) do not fall into the overweight or obese categories [35]. This discovery implies that utilizing BMI alone may not provide a comprehensive evaluation of the MAFLD risk [36]. It is important to point out that, as with previous research, we have found a strong relationship between RDW [37], ALT [38], AST, BUN, SUA [39], DBP, SBP, serum iron levels, serum potassium levels, and globulin levels with MAFLD in our study. In conclusion, these factors show promise as valuable indicators for evaluating the risk of MAFLD and predicting disease progression. A gender-specific study carried out in Japan showed the efficacy of CMI in a population that did not meet the criteria for metabolic syndrome. This result explains the expanding utility of CMI beyond the traditional at-risk group [40].

One of the key advantages of this research is its utilization of a cross-sectional design, drawing from a substantial pool of the general American adult population sampled through the complex multistage probability sampling approach of NHANES 2017–2020. The study delves into examining the connection between CMI and MAFLD, along with its subcategories. By collecting baseline data on different lifestyle factors such as alcohol intake, smoking habits, and PA, the researchers aimed to account for potential confounders in their analysis. This thorough approach not only facilitated a more in-depth interpretation of the primary findings but also allowed for a comprehensive sensitivity analysis to corroborate the results.

Nevertheless, there are constraints such as the inability to prove causality because of the observational approach, the importance of being cautious in interpreting risk estimates due to numerous comparisons that could result in false outcomes, and limitations in the database that restrict the inclusion of all factors influencing CMI and MAFLD while ensuring a large enough sample size. However, our data effectively explore the relationship between CMI and MAFLD, offering further supporting evidence on the subject.

## 5 Conclusion

Our results indicate a possible link between a higher CMI and the presence of MAFLD. This study found a curvilinear connection between CMI and MAFLD, with an inflection point identified at CMI = 0.4554. Importantly, there was a significant direct correlation between CMI and the risk of developing MAFLD. Post the inflection point (CMI = 0.4554), the rate of CMI increase slowed compared to pre-inflection levels, yet the positive association persisted. These important findings should be considered when formulating and updating clinical guidelines, although further validation and confirmation are required. However, further research is needed to investigate possible differences between populations. This will improve our understanding of the underlying mechanisms of CMI and provide valuable insights for clinical practice.

## Supplementary Material

supplementary material
