# Supplementary material for: Non-linear associations between cardiovascular metabolic indices and metabolic-associated fatty liver disease: A cross-sectional study in the US population (2017–2020)
Source: Open Life Sci. 2024 Sep 10;19(1):20220947. doi: 10.1515/biol-2022-0947 (PMC11406434; doi:10.1515/biol-2022-0947)
Supplement: supplementary material [file biol-2022-0947-sm.pdf]

Supplementary material

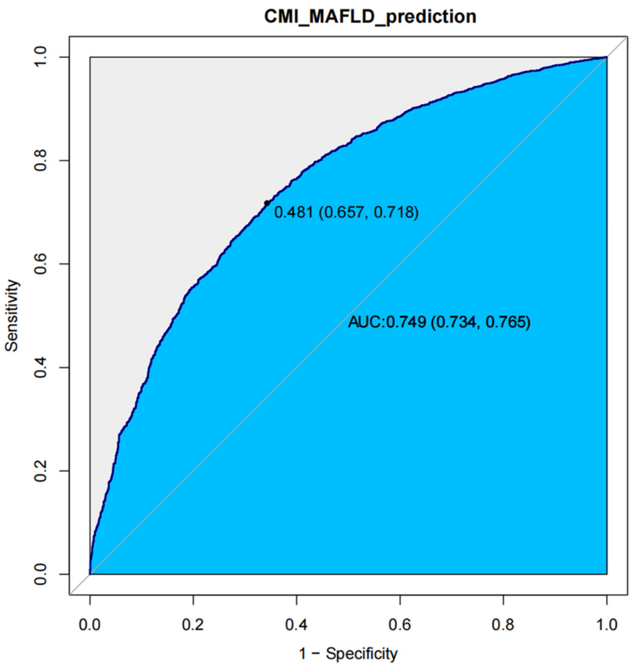

Figure S1: ROC between CMI and MAFLD. AUC is the area under the curve in blue, reflecting the predictive efficacy of CMI for MAFLD(AUC = 0.749, 95%CI: 0.734–0.765).

Table S1: Population characterisation using CMI = 0.4554 as the inflection point

Variables	Total (*n* = 3,749)	CMI ≤ 0.4554 (*n* = 1,855)	CMI > 0.4554 (*n* = 1,894)	*p*
**MAFLD, *n* (%)**				<0.001
No	1667 (44.5)	1167 (62.9)	500 (26.4)	
Yes	2082 (55.5)	688 (37.1)	1394 (73.6)	
**Gender, *n* (%)**				<0.001
Male	1820 (48.5)	812 (43.8)	1008 (53.2)	
Female	1929 (51.5)	1043 (56.2)	886 (46.8)	
**Age, Mean ± SD**	50.7 ± 17.3	48.6 ± 18.0	52.7 ± 16.3	<0.001
**Race, *n* (%)**				<0.001
Mexican American	481 (12.8)	181 (9.8)	300 (15.8)	
Other Hispanic	381 (10.2)	153 (8.2)	228 (12)	
Non-Hispanic White	1272 (33.9)	594 (32)	678 (35.8)	
Non-Hispanic Black	947 (25.3)	603 (32.5)	344 (18.2)	
Other	668 (17.8)	324 (17.5)	344 (18.2)	
**PIR, *n* (%)**				<0.001
Low	873 (23.3)	399 (21.5)	474 (25)	
Middle	1305 (34.8)	629 (33.9)	676 (35.7)	
High	1071 (28.6)	589 (31.8)	482 (25.4)	
**Education, *n* (%)**				<0.001
No	709 (18.9)	257 (13.9)	452 (23.9)	
Yes	3038 (81.1)	1596 (86.1)	1442 (76.1)	
**Married, *n* (%)**				<0.001
No	1526 (40.7)	810 (43.7)	716 (37.8)	
Yes	2219 (59.2)	1042 (56.2)	1177 (62.1)	
**Alcohol, *n* (%)**				0.751
No	3395 (90.6)	1677 (90.4)	1718 (90.7)	
Yes	354 (9.4)	178 (9.6)	176 (9.3)	
BMXBMI, Mean ± SD	29.8 ± 7.3	27.1 ± 6.2	32.5 ± 7.3	<0.001
**PA, *n* (%)**				<0.001
Low	463 (12.3)	203 (10.9)	260 (13.7)	
Middle	1682 (44.9)	890 (48)	792 (41.8)	
High	676 (18.0)	375 (20.2)	301 (15.9)	
**Smoke, *n* (%)**				<0.001
No	2133 (56.9)	1122 (60.6)	1011 (53.4)	
Yes	1614 (43.1)	731 (39.4)	883 (46.6)	
**Hypertension, *n* (%)**				<0.001
No	1647 (43.9)	980 (52.8)	667 (35.2)	
Yes	2102 (56.1)	875 (47.2)	1227 (64.8)	
**Diabetes, *n* (%)**				0.077
No	1293 (34.5)	614 (33.1)	679 (35.9)	
Yes	2456 (65.5)	1241 (66.9)	1215 (64.1)	
**Depression, *n* (%)**				0.035

Table S1: *Continued*

Variables	Total (*n* = 3,749)	CMI ≤ 0.4554 (*n* = 1,855)	CMI > 0.4554 (*n* = 1,894)	*p*
No	2828 (75.4)	1427 (76.9)	1401 (74)	
Yes	921 (24.6)	428 (23.1)	493 (26)	
WAIST, Mean ± SD	100.8 ± 17.1	93.3 ± 15.3	108.0 ± 15.7	<0.001

Table S2: Univariate Analysis of the study

Variable	OR_95CI	P_value
CMI	12.99 (9.46–17.84)	<0.001
GENDER	0.86 (0.72–1.02)	0.081
Age	1.03 (1.02–1.03)	<0.001
Other Hispanic	0.74 (0.5–1.08)	0.122
Non-Hispanic White	0.82 (0.61–1.1)	0.183
Non-Hispanic Black	0.45 (0.33–0.61)	<0.001
Other	0.5 (0.36–0.7)	<0.001
PIR:MIDDLE	1.17 (0.94–1.46)	0.17
PIR:HIGH	0.89 (0.71–1.12)	0.32
Education	0.79 (0.62–1.01)	0.064
Married	1.48 (1.24–1.77)	<0.001
Alcohol	1.37 (1.02–1.84)	0.034
BMI	1.23 (1.21–1.26)	<0.001
PA:MIDDLE	0.86 (0.67–1.11)	0.244
PA:HIGH	0.72 (0.54–0.95)	0.02
Smoke	1.31 (1.1–1.56)	0.003
Hypertension	2.92 (2.44–3.49)	<0.001
Diabetes	1.23 (1.03–1.47)	0.025
Depression	1.33 (1.08–1.63)	0.007
Waist(cm)	1.1 (1.09–1.11)	<0.001
Fasting glucose (mmol/l)	1.72 (1.55–1.91)	<0.001
Glycohemoglobin (%)	2.01 (1.75–2.32)	<0.001
Hs-CRP (mg/L)	1.08 (1.06–1.11)	<0.001
Median cap, decibels per meter (db/m)	1.33 (1.27–1.39)	<0.001
HOMA-IR	1.4 (1.33–1.47)	<0.001
